# Supplementary material for: Exploring the perceptions of the effect of the COVID-19 pandemic on the mental well-being and medical education of medical students in Northern Ireland, in addition to the perceived barriers to seeking support; a qualitative study
Source: PLoS One. 2025 Jan 9;20(1):e0314115. doi: 10.1371/journal.pone.0314115 (PMC11717186; doi:10.1371/journal.pone.0314115)
Supplement: Attachment — Submitted filename: Response to Reviewers Letter.docx [file pone.0314115.s001.docx]

Dear Editors,

In this letter I will address the comments in the decision letter regarding my previous submission to PLOS (PONE-D-23-43772). The comments from the previous decision letter are as follows:

- Based on our evaluation of your manuscript and the information you provided, we do not feel that your submission meets our ethical requirements for human subjects’ research submissions. PLOS ONE requires that research meets all applicable standards for the ethics of experimentation and research integrity (http://journals.plos.org/plosone/s/human-subjects-research). We reserve the right to reject any submission that does not meet our internal ethical standards, which in some cases are more stringent than local ethical standards.
- We recognize that you obtained ethical approval from your local ethics committee after you began your study: your ethics approval document is dated January 8, 2024, while you began collecting patient samples on 18th May 2023. You also state that informed consent was obtained from all participants, which indicates that the data were prospectively collected for research purposes. For human subjects’ research, PLOS ONE requires that authors obtain ethics committee approval before beginning the study, and we do not accept retrospective ethical approval.

In response to the comments above, there has been a misunderstanding with regards to when ethical clearance was obtained for my research. I have uploaded the official letter of ethical clearance from the Maastricht University Ethical Clearance team.

Ethical clearance was obtained via email on 17^th^ May 2023; as stated in my previous submission. I have attached a copy of this email below (figure 1). Participant recruitment then began after this approval; planning with regards to whom to contact (18^th^ May 2023) and then first contact with participant made on 24^th^ May 2023.

I contacted the ethical clearance team in January 2024 to obtain a letter of proof of ethical clearance for the PLOS one submission process, as I felt attaching the email was insufficient and somewhat unprofessional. The ethical clearance team were happy to provide an additional letter of proof, which they dated January 8^th^, 2024 (the date they wrote the letter). This led to my previous submission being rejected as PLOS thought ethical clearance was obtained after research completion. To rectify this, I contacted Maastricht University Ethical clearance in January 2024 to provide a new letter of proof of ethical clearance; providing the date they approved my research, and also the date the letter was written. I have uploaded this letter.

To further confirm the timeline of my research: research proposal and ethical clearance form completed (February – May 2023), ethical clearance obtained (May 2023), participant recruitment including obtaining written and verbal informed consent, interview process, analysis and research write up (May - August 2023).

I hope this is all clear. Please contact me or the Maastricht University Ethical clearance team if is any uncertainty.

Figure 1; Email of proof of ethical clearance provided 17^th^ May 2023

Kind regards,

Claire Whiteside^1,2^**^¶^**^,^
