# Supplementary material for: Exploring the perceptions of the effect of the COVID-19 pandemic on the mental well-being and medical education of medical students in Northern Ireland, in addition to the perceived barriers to seeking support; a qualitative study
Source: PLoS One. 2025 Jan 9;20(1):e0314115. doi: 10.1371/journal.pone.0314115 (PMC11717186; doi:10.1371/journal.pone.0314115)
Supplement: Attachment — Submitted filename: Response to reviewers letter.docx [file pone.0314115.s002.docx]

Dear Editors,

In this letter I will address the comments in the decision letter regarding my previous submission to PLOS (PONE-D-24-04972). The submission was rejected with major revisions. I have attached both a marked-up copy of the manuscript and an unmarked version of the revised manuscript. In the marked-up copy, the red and underlined words signify added material, while the red crossed out words signify deleted material. Below, the comments raised by the academic editor and reviewers from the previous decision letter are highlighted in bold with my responses underneath.

**Journal Requirements:**

**When submitting your revision, we need you to address these additional requirements.**

1. **Please ensure that your manuscript meets PLOS ONE's style requirements, including those for file naming. The PLOS ONE style templates can be found at**
   [**https://journals.plos.org/plosone/s/file?id=wjVg/PLOSOne_formatting_sample_main_body.pdf**](https://journals.plos.org/plosone/s/file?id=wjVg/PLOSOne_formatting_sample_main_body.pdf) **and** 
   [**https://journals.plos.org/plosone/s/file?id=ba62/PLOSOne_formatting_sample_title_authors_affiliations.pdf**](https://journals.plos.org/plosone/s/file?id=ba62/PLOSOne_formatting_sample_title_authors_affiliations.pdf)

I have checked the PLOS style requirements and ensured the manuscript; including tables, figures and references meet the required styles.

2. **We note that your Data Availability Statement is currently as follows:**

All relevant data are within the manuscript and its Supporting Information files.

**Please confirm at this time whether or not your submission contains all raw data required to replicate the results of your study. Authors must share the “minimal data set” for their submission. PLOS defines the minimal data set to consist of the data required to replicate all study findings reported in the article, as well as related metadata and methods (**[**https://journals.plos.org/plosone/s/data-availability#loc-minimal-data-set-definition**](https://journals.plos.org/plosone/s/data-availability#loc-minimal-data-set-definition)**).**

**For example, authors should submit the following data:**

- **The values behind the means, standard deviations and other measures reported;**
- **The values used to build graphs;**
- **The points extracted from images for analysis.**

**Authors do not need to submit their entire data set if only a portion of the data was used in the reported study.**

**If your submission does not contain these data, please either upload them as Supporting Information files or deposit them to a stable, public repository and provide us with the relevant URLs, DOIs, or accession numbers. For a list of recommended repositories, please see** [**https://journals.plos.org/plosone/s/recommended-repositories**](https://journals.plos.org/plosone/s/recommended-repositories)**.**

**If there are ethical or legal restrictions on sharing a de-identified data set, please explain them in detail (e.g., data contain potentially sensitive information, data are owned by a third-party organization, etc.) and who has imposed them (e.g., an ethics committee). Please also provide contact information for a data access committee, ethics committee, or other institutional body to which data requests may be sent. If data are owned by a third party, please indicate how others may request data access.**

This is a qualitative research study. Therefore, all data is in the form of recordings which contain identifiable and sensitive information. Apart from length of participant interviews which is included in the revised manuscript, there is no numerical data or graphs. There are anonymous quotations within the manuscript results section. Upon participant recruitment, the following information was in the participant information letter:

‘The data will be stored anonymously and be inaccessible to all excluding the principal researcher and supervising researcher. The data will be used to write up a thesis project which will be submitted to journals to be potentially published. If the opportunity arises, the results of the research will be made into a poster or PowerPoint for presentation for the potential to be presented at Maastricht University and or a national/international conference. If desired, the thesis will be made accessible to participants after completion. participants were informed all data’.

Upon applying for ethical approval from the Maastricht University Ethics committee, the following information was in the approval request letter:

‘Identifiable data, such as audio recordings and transcripts will only be accessible by the principal researcher. Anonymous data will only be accessible by the research team; made up of the principal researcher and two research supervisors. The principal researcher will act as the data controller.’

‘Other individuals will not be able to access the data as it will be sorted on a password protected file on a password protected laptop during the project. After the project it will be stored on the UM server in password protected files. The data will not be printed.’

‘The data will not be shared or used in future research. However, the thesis has the potential to be published in a journal. Therefore, the publication could potentially be reviewed in a literature review. Potential publication will be referenced in the information and consent form.’

Therefore, sharing the data would both violate the agreement with the participants prior to data collection, in addition to the agreement made upon approval for the research with the Maastricht University ethics committee. The data is currently being securely stored in encrypted files by the principal researcher. Anonymous transcripts can be obtained from the principal researcher, Claire Whiteside on request but they cannot be shared publicly. The University of Maastricht Ethics committee address is Universiteitssingel 40, 6229 ER Maastricht, Netherlands and contact email is [ethical_clearance_gh@maastrichtuniversity.nl](mailto:ethical_clearance_gh@maastrichtuniversity.nl)

3. **When completing the data availability statement of the submission form, you indicated that you will make your data available on acceptance. We strongly recommend all authors decide on a data sharing plan before acceptance, as the process can be lengthy and hold up publication timelines. Please note that, though access restrictions are acceptable now, your entire data will need to be made freely accessible if your manuscript is accepted for publication. This policy applies to all data except where public deposition would breach compliance with the protocol approved by your research ethics board. If you are unable to adhere to our open data policy, please kindly revise your statement to explain your reasoning and we will seek the editor's input on an exemption. Please be assured that, once you have provided your new statement, the assessment of your exemption will not hold up the peer review process.**

This research is unable to adhere to the open data policy, therefore the principal researcher requests an exception. The data cannot be shared publicly because of the initial agreement with participants and Maastricht University ethics committee as described above. Participants were informed all data would be anonymised and only available to the principal researcher and supervising researcher. The qualitative data is available from the Claire Whiteside; the principal researcher on request; for researchers who meet the criteria for access to confidential data.

**Additional Editor Comments:**

**In addition to reviewers' comments, please address my below comments:**

1. **Please indicate the study design in the abstract. Did you follow a descriptive design? Or any other study design? Indicate the analysis technique in the abstract.**

The methods section in the abstract has been extended to include additional study design information and analysis technique.

2. **In the abstract, results lack substances. For example, you talked about impact without indicating the impacts of covid. Please provide more details of impact for the readers.**

The results section of the abstract has been extended to include additional information.

3. **Reduce the number of objectives and mention the objective you addressed and indicated in the paper title/abstract. Or change the title. This means there should be consistency between your paper aim, title and results you described. The current title only indicates the impact. But you described barriers etc. Make necessary changes everywhere.**

The title has been changed to more appropriately encompass the four study objectives. There is now consistency throughout the manuscript.

4. **Analysis technique is absent. Please detail.**

Section 3; methods has been expanded to include more information regarding study design, participant demographics and data analysis technique.

5. **Please summarise your findings in the discussion section before comparing them with other studies.**

An additional paragraph has been added in section 5 to summarise the research findings prior to comparing the results with other studies.

**Reviewers Comments:**

**Reviewer #1:**

**This is a qualitative study article on the effect of COVID-19 on the mental well-being of medical students in Northern Ireland. The discussion on the basics is fine and based on a broad understanding of the field. However, it seems to need to with a few sentences in the introduction by focusing more on Northern Ireland during the COVID-19 time to distinguish it from other similar studies (Prevalence rate, government services, accessibility to vaccine...). Also, let me know why the authors decided to interview participants three years after COVID-19. How are authors sure in these years participants were not involved with other emotional damage?**

Section 1; the introduction has been adjusted to include more information relevant to COVID-19 in Northern Ireland, such as prevalence, death rates and vaccine uptake.

The participants were interviewed in June 2023. The COVID-19 pandemic initiated in Northern Ireland in March 2020 and all restrictions has ceased 2 years later in March 2022. Therefore, there was a 15-month gap between complete cessation of the pandemic in NI and interviewing participants. The principal researcher decided to complete the research at this point because this is when they were completing a masters in Global Health at Maastricht University. The research was carried out for the purpose of their master’s thesis. The principal researcher felt this was an appropriate time to complete this retrospective qualitative research as participants would be able to recall the effects of the pandemic on their mental health during the pandemic, as well as perceived secondary effects on their medical education; both during the pandemic and at the time of data collection.

**Reviewer #2: Thank you for the paper**

- **Abstract needs revision. I do not know how the authors mentioned significant results in qualitative. This section needs revision - Methods part needs comprehensive revision**
- **Add rigor in Method**
- **In results, show themes, subthemes and code**
- **Add demographic characteristics of the study sample**
- **Revise discussion and link to results**

The abstract has been revised, with the word ‘significant’ removed and more detail in method section. The results section has been changed, with added adjusted theoretical frameworks (figure 1 and 2) to demonstrate themes, subthemes and code. Table 1 has been added to demonstrate the demographics of the study sample. The discussion has been revised with added summary paragraph.

**Reviewer #3:**

1. **I think, your paper is well written, but it is more suitable for PLOS Mental Health Journal.**

I will consider applying to the PLOS mental health journal if the manuscript is not successful in PLOS One.

2. **Line number 135: "The principal researcher/author is a female Northern Irish medical student studying..."
   why is it necessary to mention the gender of the principal researcher/author?**

The gender of the principal researcher has been removed.

3. **Line number 151: "... average length being 30.4 minutes."
   I think Standard deviation should be included here in order to understand the dispersion/spread of the interview length.**

Standard deviation has been added and interview lengths are demonstrated in table 1.

4. **Line number 593: "This was the first research carried out in NI assessing barriers mental health support utilisation amongst...."
   If you are 100% sure about what has been written, then it is alright... Congratulation on your work. But if not, please write as follows: "as far as we know" or "to our knowledge". In that case, it would not create any possible conflict of interest in future.**

Necessary adjustments have been made to reduce opportunity for possible future conflicts of interest.

5. **Is NHS under the jurisdiction of Northern Ireland? or it is HSC that is under the jurisdiction of NI? I am pointing out it as the UK NHS has been mentioned in Line number 105. NI may have different publicly governed/funded healthcare system if I am not wrong.**

The NHS is the National Health service of the UK; including Northern Ireland. There are different branches of the UK health system. NHS England is centrally funded from the Department of Health and Social care while Northern Ireland receives a grant from the UK treasury which funds the Department of health, social services and public safety for Northern Ireland (DHSSPS). The slight difference between health systems in England and NI is that both the health and social care are provided through an integrated service. Therefore, Health and social care Northern Ireland (HSCNI) is part of the overall UK NHS. The sentence (now line 155) has been adjusted to more appropriately reflect this.

Please let me know if any additional clarification is required for any of the editors or reviewers’ comments.

Kind regards,

Claire Whiteside^1,2^**^¶^**^,^
